# Insights into the Mechanism of Bipolar Electrodeposition of Au Films and Its Application in Visual Detection of Prostate Specific Antigens

**DOI:** 10.3390/bios13020158

**Published:** 2023-01-19

**Authors:** Daoyuan Zhao, Yujing Liu, Hong Jiang, Haijian Yang, Huihui Yu, Jingtang Qiao, Zhiwen Li, Bing Jin, Meisheng Wu

**Affiliations:** 1 Department of Chemistry, College of Sciences, Nanjing Agricultural University, 1 Weigang, Nanjing 210095, China; 2 College of Life Sciences, Nanjing Agricultural University, 1 Weigang, Nanjing 210095, China

**Keywords:** bipolar electrode, ECL imaging, electrochemical imaging, electrochemical dissolution, biosensor

## Abstract

Au particles are commonly used for deposition on the surface of a bipolar electrode (BPE) in order to amplify electrochemical and electrochemiluminescence (ECL) signal because of their excellent conductivity, biocompatibility, and large surface area. In this work, a closed BPE device was fabricated and Au particles were deposited on the two poles of a BPE via bipolar deposition. Results indicated that the electrochemical stability of Au film on the anode part of the BPE and the reduction of AuCl_4_^–^ to Au on the cathode part of the BPE depended on the conductivity of the solution. The prepared Au–Au BPE exhibited a remarkable amplification effect on the ECL signal. Then, a specific sensing interface was constructed on one pole of the BPE for the visual detection of prostate-specific antigens (PSA) based on sandwich-type immunoreactions between primary PSA antibodies (Ab_1_) on the electrode surface, PSA, and SiO_2_ nanoparticles labeled secondary PSA antibodies (SiO_2_-Ab_2_). The designed biosensor exhibited a good linear relationship for the ECL detection of PSA in the range of 1 × 10^−6^ to 1 × 10^−10^ g/mL with a correlation coefficient of 0.9866; the limit of detection (LOD) was 1.5 × 10^−11^ g/mL. Additionally, the biosensor can realize the electrochemical imaging of PSA by regulating the electrochemical oxidation of the Au anode with the immunoreactions on the cathode part of BPE. Therefore, the small, portable and highly sensitive biosensors have great potential for on-site detection.

## 1. Introduction

Bipolar electrochemistry devices, as a newly emerging analytical approach, have attracted significant attention because of their easy fabrication, high sensitivity, low production cost, etc. [1,2,3,4,5,6]. A bipolar electrode is a conductor situated in solution; it has no direct electrical connection with an external power source. When an electric field is applied to the solution, a potential difference is generated between the conductor and the solution on its surface. Therefore, one end of the conductor becomes the anode, and the other end becomes the cathode, namely bipolar electrodes (BPEs). According to the current flow path, BPE devices can be divided into open BPEs and closed BPEs [7,8]. In closed BPEs, the anode and the cathode of the BPE are immersed into two electrochemical cells which enable the specific modifications of one pole of the BPE and avoid the chemical interference between the two solutions.

In recent years, visualized detection of the current changes in BPEs using imaging technology has attracted great attention [9,10,11,12]. For example, Liu et al. constructed a multicolor electrochemiluminescence (ECL)-BPE device for visualized sensing of Salmonella typhimurium [13]. Cheng has developed a novel ratiometric ECL-BPE device for miRNA-21 measurement by introducing mesoporous silica nanoparticles (NPs) at the cathode of the BPE as a molecular gating system to amplify the cathodic ECL signal [14]. To achieve a visual readout of target, it is essential to integrate signal amplification strategies into the BPE system.

Since the only way that the current can pass through the two electrochemical cells in closed BPE devices is by BPE, many efforts have been made to improve the electrochemical signal at the anode of BPEs, such as changing the electrochemical reactions that occur at the cathode of BPEs [11] and introducing electroactive species and catalysts on the cathode of BPEs [15,16,17] to increase the current [18], catalyze the redox reactions of the BPEs [19], and decrease the onset voltage for driving the redox reactions occurring at BPEs [20,21]. For example, Xu and coworkers have developed an enhancement strategy to detect prostate-specific antigen (PSA) through a BPE-ECL approach [22]. By immobilizing the thionine@ SiO_2_ NPs at a BPE’s cathode and the Au nanoparticles at its anode through DNA hybridization, the ECL signal was improved approximately five-fold compared with that of a bare indium tin oxide (ITO) electrode.

Besides the above catalysts, Au NPs are extensively used in BPE devices due to their good electrical conductivity, high catalytic activity, and large specific surface area [5,16,23,24]. For example, Ge and coworkers have constructed an Au BPE by growing an Au NPs layer in situ on the surfaces of cellulose fibers to improve the BPE’s conductivity [23]. In addition, they constructed a biosensing interface with AuPd NPs tags at the cathode of the bipolar electrode which can catalyze a H_2_O_2_ reduction reaction, causing significant enhancement of the ECL signal at the anode. The designed biosensor enables the measurement of miRNA-155 in the range of 1 pM–10 μM, with a detection limit of 0.67 pM. Ding’s group introduced patchy gold coated Fe_3_O_4_ hybrid nanoparticles on Au NPs-modified cathode in immunoreaction [24]. The biosensor achieved sensitive detection of carcinoembryonic antigen (CEA) with a low detection limit of 0.03 pg/mL. However, little attention has been paid to improve the sensitivity of BPE-ECL device by modifying Au NPs on both poles of BPE. This is because Au NPs on the anode of BPE may undergo electrochemical oxidation under high voltage, thereby reducing the stability and reproducibility of the BPE. Therefore, improving the stability of the gold film in the BPE system is one of the challenges in further improving the sensitivity of BPE systems.

Inspired by these, we designed a portable ECL-BPE device and attempted to fabricate a Au (cathode)-Au (anode) BPE through bipolar deposition. The oxidation conditions of the Au anode and the deposition conditions of the Au particles on the cathode of the BPE were studied in detail by adjusting the solution in the anodic reservoir. The amplification effect and the reproducibility of the prepared BPE were evaluated by ECL imaging. Then, a sandwich-type immunointerface was constructed on the cathode of the BPE by using SiO_2_-Ab_2_ as the recognition probe. In the presence of PSA, SiO_2_-Ab_2_ was bound to electrode surface, leading to an inhibited ECL signal at the anode of the BPE due to the increased resistance. Additionally, the PSA could be visualized using the electrochemical imaging method based on the resistance-modulated oxidation of the Au film on the anode.

## 2. Materials and Methods

### 2.1. Reagents

Chloroauric acid (HAuCl_4_), N-(3-(Dimethylamino)propyl)-N’-ethylcarbodiimide hydrochloride (EDC), N-hydroxysuccinimide (NHS), and Ru(bpy)_3_^2+^ were purchased from Sinopharm Chemical Reagent Company (Shanghai, China). L-Cysteine was purchased from Sinopharm chemical reagent company (Shanghai, China). Total prostate-specific antigen (PSA) and PSA antibodies were purchased from Linc-Bio Science (Shanghai, China). SiO_2_ nanoparticles modified with KH550 (20 nm) were purchased from Nanjing Xianfeng Nano Co. (Nanjing, China). A 10 mM PBS buffer solution (pH 7.4) was purchased from KeyGEN Bio-Technology Co., Ltd. (Taixing, China). Sylgard 184 (including a polydimethylsiloxane (PDMS) monomer and curing agent) was purchased from Dow Corning (Midland, MI, USA). Graphite paper (GP) was purchased from Jing Long TeTan Co., Ltd. (Beijing, China).

### 2.2. Instruments

The voltage applied on the driving electrodes was supplied by a CHI 660E electrochemical workstation (Shanghai Chenhua instrument, Shanghai, China). A MS23 CCD camera was used to capture images and movies (Guangzhou Mingmei Technology Co., Ltd., Guangzhou, China). The conductivity meter used (DDS-307A) was purchased from INESA Scientific Instrument Co., Ltd., Shanghai, China.

### 2.3. Bipolar Deposition

A closed BPE device was designed by placing graphite paper (GP, length of 2 cm, width of 1 cm) on a flat PDMS slice and then covering it with two layers of PDMS slices (Scheme S1). Two holes were punched in each PDMS slice as reservoirs. The diameter of holes on the second PDMS slice was 3 mm, with a gap of 2 mm. The diameter of the holes on the top layer was 7 mm, with a gap of 1 mm. After that, two Pt wires were placed on the top of the second PDMS slice and connected with CHI 660E to perform the bipolar deposition of Au.

Bipolar deposition of Au particles at the two poles of BPE was carried out in two steps. First, 100 µL of 5 mM HAuCl_4_ was introduced into the right reservoir and 100 µL of PBS (10 mM, pH 7.4) was added into the left reservoir. A constant voltage of 4.5 V was applied for 300 s, using an amperometric i-t curve technique. After finishing the deposition, the solutions were removed and the GP was washed with water three times. Second, the direction of the electric field was reversed. The left reservoir was filled with 5 mM HAuCl_4_ and the right reservoir was filled with different solutions such as water, various concentrations of PBS, KCl, HCl, and HAuCl_4_. A constant voltage of 4.5 V was applied for 300 s. Movies and images were taken by a CCD camera at the same time. In order to study the mechanism, a linear sweep voltage (LSV) from 0 to 5.5 V at a scan rate of 0.005 V/s was applied to the driving electrodes. Images were taken every 1 min and when the current exhibited remarkable change.

### 2.4. Fabricating of Specific Sensing Interface for PSA Assay

The left pole of GP BPE was first modified with Au particles by adding 100 µL of 5 mM HAuCl_4_ in the left reservoir, PBS in the left reservoir and a constant voltage of 4.5 V for 300 s. Then, the right pole of the BPE was modified with Au particles by applying 4.5 V for 200 s, and the two reservoirs were filled with 5 mM HAuCl_4_. The left reservoir was filled with 0.175 M L-cysteine at room temperature for 10 h. Then, the carboxyl group at the Au surface was activated by the mixture solution of 7.0 mg/mL EDC and 21.0 mg/mL NHS. After rinsing with PBS, 20 µL of primary PSA antibody (Ab_1_) was introduced and incubated at 4 °C overnight. Then, 0.2% BSA was added to block the unreacted sites on Au NPs at 37 °C for 1 h in order to avoid non-specific adsorption. The modified electrode was then incubated with different concentrations of PSA. After washing with PBS, 20 µL of SiO_2_-modified secondary PSA antibody (SiO_2_-Ab_2_) was introduced and reacted at 37 °C for 1 h. Finally, the electrode was washed with PBS, and a sandwich immunocomplex was formed at the right pole of BPE.

### 2.5. ECL Imaging

The left reservoir was filled with 50 mM H_2_O_2_ (PBS) and the right reservoir was filled with the mixture solution of 10 mM Ru(bpy)_3_^2+^ and 100 mM 2-(Dibutylamino) ethanol (DBAE). A constant voltage of 3.5 V was applied and the ECL images were captured by the CCD camera. The exposure time was 7 s.

### 2.6. Electrochemical Imaging

For the visual assay of PSA based on the oxidative electrodissolution of the Au anode, the left reservoir was filled with 100 μL of 50 mM H_2_O_2_ (PBS) and the right reservoir was filled with 100 µL of PBS. The external voltage was set at 3.0 V to drive the dissolution of Au film in the right reservoir.

## 3. Results and Discussion

### 3.1. Bipolar Deposition of Au on Two Poles of BPE

In our study, both ends of a graphite paper (anode and cathode) were exposed to different solutions; this can effectively avoid the chemical interference of the solutions. Through this configuration, different interfaces can be constructed on the anode and cathode of the device. For example, a signal amplification unit can be constructed at one end, and a sensing interface can be constructed at the other end for the specific detection of target substances. Gold was electrodeposited on both ends of the bipolar electrode by two-step electrodeposition. First, Au particles were deposited on one end of the electrode by bipolar electrodeposition. Then, the direction of the applied electric field was changed, and Au particles were deposited at the other end. At the same time, the factors affecting the electrochemical stability of gold films and the electrodeposition of gold were investigated. Finally, the Au (cathode)-Au (anode) bipolar electrode was used for the visualization of prostate-specific antigen (PSA).

**Figure 1:**
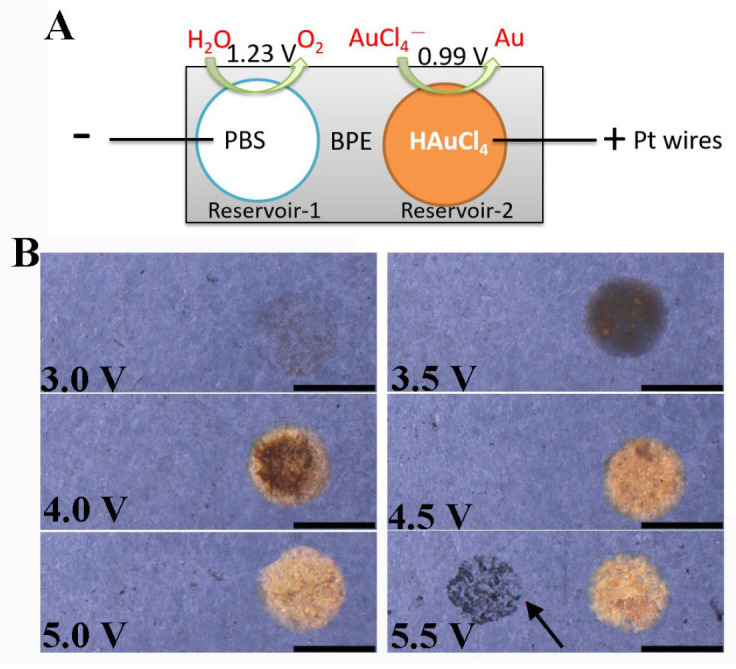
Bipolar electrodeposition of Au particles on one pole of the GP BPE. (**A**) Schematic representation of bipolar deposition of Au on one pole of GP BPE. (**B**) Optical images of Au film obtained under various external voltages from 3.0 to 5.5 V for 300 s. The concentration of HAuCl_4_ was 5 mM. The black arrow indicates the corrosion of GP in the right reservoir; the scale bar represents 3 mm.

The detailed construction process of the closed bipolar electrode device was described as the following. Two Pt wires were placed on the top of the middle PDMS slice and were connected to a power source. We plated Au particles at the GP surface in the right reservoir, under voltages ranging from 3.0 to 5.5 V, by filling reservoir-1 (anode) with 10 mM PBS, and reservoir-2 (cathode) with HAuCl_4_ (Figure 1A). The reduction of HAuCl_4_ at the BPE cathode in reservoir-2 was coupled with the oxidation of H_2_O at the BPE anode in reservoir-1. Figure 1B shows that the GP color (in reservoir-2) gradually turned yellow with the increase in external voltage. However, the left reservoir’s GP was damaged (as pointed out by the red arrow) when the voltage was 5.5 V. Additionally, a lot of bubbles could be observed escaping from the Au surface due to the reduction of H^+^, when the voltage was 5.0 V, which resulted in the formation of a porous Au film. Therefore, 4.5 V was chosen as the optimal voltage for the bipolar deposition of Au.

**Figure 2:**
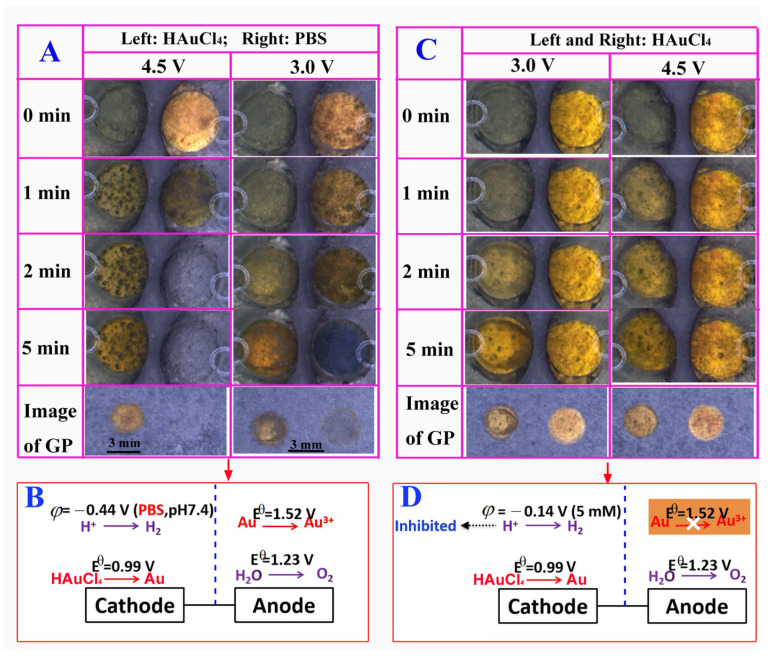
Deposition of Au particles on GP in the left reservoir. GP in the right reservoir was pre-modified with Au particles under 4.5 V for 300 s through bipolar deposition. (**A**) The left reservoir was filled with 5 mM HAuCl_4,_ and the right reservoir was filled with 10 mM PBS. (**B**) Redox reactions occurred at the two poles of the BPE in (**A**). (**C**) Both reservoirs were filled with 5 mM HAuCl_4_. (**D**) Redox reactions occurred at the two poles of the BPE in (**C**). The deposition time was 300 s.

After washing the prepared Au-modified BPE with water, we reversed the direction of the electric field and exchanged the solutions in these two reservoirs for the deposition of Au film on the left GP surface. We applied 4.5 V to the driving electrodes for 5 min. Figure 2A and Video S1 (×5 frame rate) show that Au particles were produced in the left reservoir with a lot of bubbles, indicating that both AuCl_4_^−^ and H^+^ were reduced at the BPE cathode (see Figure 2B for the reactions). For the anodic pole (right), water was oxidized before the Au film, because the voltage needed to drive the oxidation of water is lower than that of the Au film (Figure 2B). However, the results of Figure 2A revealed that the Au film was promptly decomposed, it disappeared within 93 s (Video S1). Once the Au completely disappeared, the current was significantly decreased and then reached a steady state (Figure S1, curve a, in Supplementary Material). This result suggested that 4.5 V was too high. We then decreased the external voltage to 3.0 V (Figure 2A). Unfortunately, the Au film was dissolved again. Additionally, the speed of Au particles’ disappearance and the number of bubbles produced at the cathode’s surface also decreased.

The above results showed that it is difficult to electrodeposit Au particles on both ends of the bipolar electrode. However, in the previous work, we successfully adopted a two-step bipolar electrodeposition method to electrodeposit gold and platinum on its two ends, using carbon spheres as the BPE [25]. Compared with the current method, an open system was used in previous work wherein both the anode and cathode of the BPE were immersed in the same solution for electrodeposition. In the current work, the end of the BPE that needed to be gold-plated was immersed in HAuCl_4_, and the anode of the BPE that did not need to be gold-plated was immersed in PBS. To understand whether the solution had an impact on the BPE’s electrochemical reactions, we replaced the PBS in the right reservoir (anode) with HAuCl_4_ during the second bipolar deposition process (Figure 2C). To our surprise, the Au film in the right reservoir showed no significant change, and bright yellow Au could be observed on the GP surface in the left reservoir (cathode) after 2 min. Even when a high voltage was applied (4.5 V) (Figure 2C, Video S2, ×10 frame rate), the Au film at the right reservoir (anode) still maintained its original color, and the Au deposition rate in the left reservoir (cathode) increased. By comparing the videos (Videos S1 and S2) and images (Figure 2A,C) captured at 4.5 V, it was found that a lower number of bubbles were produced on the cathodic BPE filled with HAuCl_4_ than those filled with PBS. This difference indicated that the reduction of protons at the cathode and the oxidation of Au at the BPE anode were greatly inhibited (Figure 2D) when PBS was replaced with HAuCl_4_. In this case, the fact that only water could be oxidized at the BPE anode suggested that the voltage at both the BPE poles was decreased insufficiently to drive the oxidation of Au at the anode. Furthermore, the amperometric i–t curve obtained from electrodes exposed to HAuCl_4_ was very stable after approximately 40 s (curve b in Figure S1), which was different from that obtained from the electrode exposed to PBS (curve a in Figure S1).

**Figure 3:**
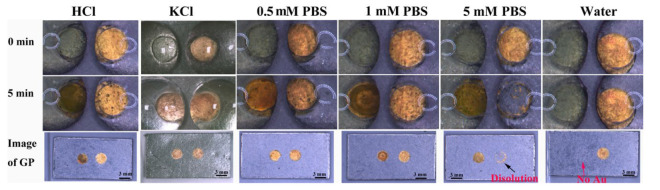
Deposition of Au at the cathode in the left reservoir. The anode was pre-deposited with Au at 4.5 V for 300 s. The left reservoirs were filled with 5 mM HAuCl_4_. The right reservoir was filled with 5 mM HCl and 5 mM KCl, respectively. The right reservoir was filled with ultra-pure water and different concentrations of PBS. Solutions were prepared with ultra-pure water unless otherwise specified.

We speculated that the factors that influence the deposition procedure included the pH and the conductivity of the solution. To verify our speculation and understand the influence of these factors on the electrochemical reaction on BPE, we replaced the right liquid reservoir with a series of different candidate solutions to carry out the next experiment. HAuCl_4_ solution was used in the left reservoir in the experiment. To investigate the effect of pH, the second-step electrodeposition was performed by using HCl solution (acidic) and KCl solution (neutral) at the right reservoir. Figure 3 shows the images of Au deposition in the second step captured before (0 min) and after (5 min). When the right reservoir was filled with either HCl or KCl solution, the colors of the Au film exhibited no significant change (at both 0 and 5 min), and Au was successfully deposited on the left GP surface (Figure 3). The result suggested that the oxidation of Au observed previously was not caused by the pH value.

Different concentrations of PBS solutions were used to study the effect of solution conductivity on the electrochemical reaction on the bipolar electrodes (Table S1). The right reservoir was filled with water, 0.5 mM PBS, 1 mM PBS, and 5 mM PBS. The left reservoir was filled with a 5 mM HAuCl_4_ solution. As shown in Figure 3, Au was deposited on the left GP without any significant change from the right reservoir’s Au film when 0.5 and 1 mM PBS were used. However, the Au film in the right reservoir decomposed when the PBS concentration was increased to 5 mM. When the right reservoir was filled with ultrapure water, no obvious change was observed on the graphite paper electrodes at the left and right ends, suggesting that the electrochemical oxidation of gold did not occur at the BPE anode, and the reduction of HAuCl_4_ did not occur at the cathode. In summary, the electrochemical reactions on bipolar electrodes are mainly affected by the conductivity of the solution.

### 3.2. Mechanism of Au Film Dissolution in BPE Device

**Figure 4:**
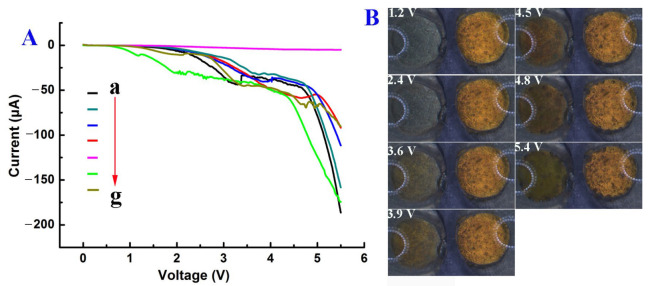
(**A**) LSV curves recorded during the second-step deposition of Au. The currents were obtained by filling the right reservoir with different solutions: 10 mM PBS (a), 5 mM PBS (b), 1 mM PBS (c), 0.5 mM PBS (d), water (e), 5 mM HAuCl_4_ (f), and 5 mM HCl (g). The left reservoir was filled with 5 mM HAuCl_4_. (**B**) Optical images were recorded during the second deposition of Au under linear sweep voltage. Both reservoirs were filled with 5 mM HAuCl_4_. The applied voltage was changed from 0 to 5.5 V at a scan rate of 0.005 V/s.

To further investigate the dissolution and formation of Au at the BPE’s two poles, a linear sweep voltage (LSV, Figure 4A), from 0 to 5.5 V was applied to the driving electrodes during the second Au deposition, and a low scan rate of 0.005 V/s was used to decrease the speed of the electrochemical reactions, including the oxidization speed of Au particles at the anode of the BPE and the reduction speed of AuCl_4_^−^ at the cathode of the BPE. With a decreased concentration of PBS, the electrical conductivity was clearly decreased (Table S1), leading to a decreased current flowing across the BPE (Figure 4A), and a high *iR_solution_* which cannot be ignored. The voltage on the BPE (ΔE_BPE_, Equation (1)) decreased accordingly, and an enhanced external voltage was needed to trigger the BPE’s electrochemical reactions.

(1) $$\Delta E_{external}=\;\;iR_{solution}+\;\;\Delta E{\;}_{BPE}+\Delta E_{sys\;}\;\;\;\;\;\;\;\;\;\;\;\;\;\;\;\;\;\;\;\;\;\;\;\;\;\;\;\;\;\;\;\;\;\;$$

where Δ*E_external_* is the voltage applied to the driving electrodes, *iR* (Δ*E_BPE_)* refers to the voltage applied at the BPE’s two poles, and *i* stands for the current flowing through the BPE. Δ*E_sys_* denotes the voltage losses in this device.

As discussed above, when the right reservoir was filled with a poor conductive solution (i.e., water), no obvious change was observed at either pole of BPE (Figure S2) and an ultra-low current was obtained (Figure 4A, curve e). If the right reservoir contained 10 mM PBS (i.e., an excellent conductive solution), the Au film started to dissolve when the voltage reached 2.9 V (Figure S3). Subsequently, the rate of dissolution increased, and most of the Au film was dissolved when the voltage was 3.4 V, leading to a sharp decrease in current (Figure 4A, curve a). The rate of Au particles’ deposition at the cathode increased along with the increase in voltage. When the voltage was further increased to 4.8 V, the GP in the right reservoir began to degrade. A small number of bubbles started to appear at the cathode’s surface when the voltage was approximately 5.2 V. A large number of bubbles at the cathode’s surface appeared when a constant voltage of 4.5 V was applied for 1 min (Figure 2A). This suggested that the formation of Au particles at the cathode could significantly catalyze the reduction of H^+^ at its surface. As the concentration of PBS was increased, the onset voltage for the deposition of Au on the GP in the left reservoir was decreased from 4.2 to 3.9 V (Figure S4). Moreover, the dissolution of the Au anode in the right reservoir was inhibited.

When the right reservoir was filled with HAuCl_4_, which is less conductive than 5 mM PBS (Table S1), the current was the highest at the lowest external voltage (around 0.5 V, Figure 4A, green line). A small amount of Au could be seen on GP in the left reservoir when the voltage was 2.4 V (Figure 4B), which is a much lower voltage than that for all the other solutions. This result illustrated that the BPE’s electrochemical reactions not only depended on the solution’s conductivity but also changed with the reactions that happened on the driving electrodes, which could enhance the current flowing through the electric circuit. The Au film in the right reservoir remained intact except for several bubbles being produced. Similar results were observed when HAuCl_4_ was replaced with 5 mM HCl. Figure S4 shows that the minimum voltage for driving the deposition of Au was about 3.3 V when the Au anode was immersed in HCl, which was lower than that in the diluted PBS solution (4.2 to 3.9 V), but was higher than that in the solution of 5 mM HAuCl_4_ (2.4 V, Figure S3) even though these solutions have similar conductivity (Table S1). This was because both the protons and AuCl_4_^−^ could be reduced at the driving electrodes in the right reservoir, leading to an enhanced current flowing through the BPE (Figure 4A). As a result, the voltage drop across the BPE increased, and a low external voltage could be applied to drive the redox reactions on the BPE. Therefore, the unwanted decomposition of the Au film at the BPE anode could be avoided by adjusting the conductivity of the solution and controlling the electrochemical reactions at the driving electrode.

### 3.3. Characterization of Au–Au BPE

Figure S5 displays the scanning electronic microscopy (SEM) images of Au film obtained under optimal conditions by filling the two reservoirs with HAuCl_4_ for bipolar deposition of Au film. Figure S5A showed that the graphite paper (GP) had a relatively smooth surface. After the first-step deposition of Au, the GP was coated with a high density of Au layer (Figure S5B). In the second-step deposition of Au, a highly dense Au layer could also be observed on GP surface (Figure S5C). The crystal structure of Au film was analyzed by XRD (Figure S6). Both Au-1/GP and Au-2/GP showed a new obvious peak at 2θ of 38.12° compared to that of GP, corresponding to the Au [111] plane [26].

**Figure 5:**
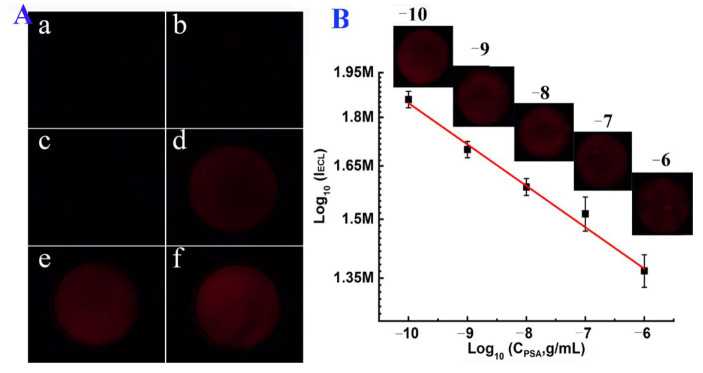
(**A**) ECL images obtained at bare (cathode)-bare (anode) GP BPE (BPE-1, a and b), bare (cathode)-Au-modified (anode) GP BPE (BPE-2, c and d), and Au-modified (cathode)-Au-modified (anode) GP BPE (BPE-3, e and f). The voltage applied in a, c, e was 3.0 V. The voltage applied in b, d, and f was 3.5 V. Images were obtained after applying voltages for 110 s. (**B**) Calibration curve for PSA detection. The deposition time of Au in the second process was 100 s. The concentration of PSA was 10^−10^, 10^−9^, 10^−8^, 10^−7^, and 10^−6^ g/mL.

The remarkable signal amplification effect of the modified BPE was then studied by immersing the Au film anode in Ru(bpy)_3_^2+^/DBAE solution and the Au film cathode in H_2_O_2_ (PBS). As shown in Figure 5A, three different BPEs, including a bipolar electrode with two poles without gold plating (BPE-1, a and b), a bipolar electrode with one pole plated with gold (BPE-2, c and d), and a bipolar electrode with two poles plated with gold (BPE-3, e and f), were used in the experiment. BPE-1 and BPE-2 were used as control electrodes. When the voltage was 3.0 V, ECL signal could only be observed on BPE-3. With the further increase in voltage to 3.5 V, the brightest ECL signal could be observed on BPE-3, indicating a high amplification effect of Au particles on the ECL signal. Additionally, Figure S7 shows the stability of ECL signal on BPE-3 under different voltages from 2.5 to 4.0 V. A stable and strong ECL intensity could be obtained when the voltage was 3.5 V. The reproducibility and stability of the prepared BPE was evaluated by recording the ECL signals on three different batches of electrodes. Each electrode was measured three times (Figure S8). The relative standard deviation (RSD) values obtained on each electrode in three consecutive measurements were 2.1%, 1.8%, and 1.3%, respectively. The RSD value on these three Au-modified (cathode)-Au-modified (anode) GP BPEs was 3.5%, indicating the good repeatability and reproducibility of the prepared BPEs. Furthermore, after three consecutive measurements, there was no significant change to the anode surface of the BPE (Figure S9). All these results showed that the prepared BPEs have good stable and signal-amplifying characteristics which are promising for sensitive ECL detection.

### 3.4. ECL Imaging of PSA

The prepared BPE device was then used for ECL detection of prostate-specific antigen (PSA) concentration based on the sandwich immunoassay. First, a primary PSA antibody was constructed at the cathode surface of Au–Au-modified GP BPE. In the presence of PSA, silica nanoparticles labeled secondary PSA-antibodies (Ab_2_-SiO_2_) (TEM image in Figure S10A, ca. 20 nm) could be captured, and formed sandwich immunocomplexes at the Au anode surface, resulting in the enhanced resistance of electrode and a quenched ECL signal. The resistance change in the presence of PSA was characterized by an electrochemical impedance spectroscopy (EIS) (Figure S10B). The resistance of bare GP was about 1600 Ω (curve a). After the deposition of Au film and the modification of primary antibody, the resistance decreased significantly (curve b) due to the excellent conductivity of Au film. When PSA was introduced, the resistance was increased (curve c). With the further combination of Ab_2_-SiO_2_, the resistance was increased again (curve d), indicating the successful formation of a sandwich structure on the electrode surface.

Figure 5B is the calibration curve of the prepared electrode for the detection of PSA at the anode of BPE. A good linear relationship was observed between the ECL intensity and the logarithmic PSA concentration from 1 × 10^−10^ to 1 × 10^−6^ g/mL, with a correlation coefficient of 0.9866. The linear equation is lg I_ECL_ = −0.0322 lgC (g/mL) + 5.944. The limit of detection (LOD) and quantification (LOQ) was calculated to be 1.5 × 10^−11^ and 1.0 × 10^−10^ g/mL based on 3 σ/s and 10 σ/s, respectively. Here, σ and s are the standard deviation of the blank signal and the slope of the calibration curve. Compared with other PSA biosensors based on imaging strategies [27,28,29,30], the ECL biosensor developed in this work displayed a wider linear range and a high sensitivity for imaging of PSA (Table S2).

The selectivity of the designed biosensor was then evaluated in the presence of interfering proteins such as thrombin and BSA. Figure S11 exhibited that the ECL signal obtained in the presence of those two proteins was similar to the blank sample, which demonstrated the biosensor has good specificity and anti-interference ability.

### 3.5. Electrochemical Imaging of PSA

**Figure 6:**
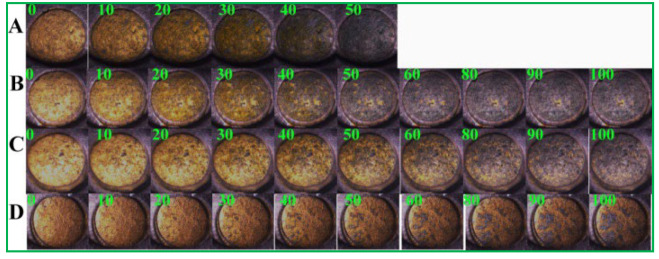
Dissolution of Au film at the anode of the BPE when the cathode of the GP BPE was modified with Au (**A**), Ab_1_/Au (**B**), SiO_2_-Ab_2_/PSA (10 ng/mL)/Ab_1_/Au (**C**), and SiO_2_-Ab_2_/PSA (100 ng/mL)/Ab_1_/Au (**D**). The external voltage was 3.0 V.

Alternatively, the electrodissolution of Au film at the anode of the BPE provided a novel way to visualize reactions at the cathode of Au/BPE. Here, the primary PSA antibody (Ab_1_) was labeled at the cathode of the BPE. Figure 6A shows that the Au anode was dissolved completely within 50 s and without modification. Upon the combination of the primary PSA antibody (Ab_1_) at its surface (Figure 6B), the oxidative dissolution time of the Au anode was prolonged slightly. After the specific formation of a sandwich immunocomplex in the presence of 10 (Figure 6C) and 100 ng/mL PSA (Figure 6D), the dissolution of the Au anode was inhibited, apparently due to the increased resistance of the Au cathode (Figure S10B). The Au film was partially dissolved, even when the voltage was applied for 100 s (Figure 6D). The change in RGB value could be used for the quantitative measurement of PSA concentration based on the dissolution of Au film. The visual readout of the dissolution of the Au anode provides a good way to observe the sensing reactions at the BPE cathode.

## 4. Conclusions

In conclusion, we studied the mechanism of electrochemical oxidation of Au particles on the anode surface of a BPE. Additionally, the factors that affect the electrodeposition of Au particles at both ends of a BPE were also studied. The electrochemical oxidation of Au film on BPE anode is affected by many factors, including solution conductivity and pH value. The Au BPE device prepared by this method showed a good signal amplification effect and can emit a strong visible red-light emission signal in ECL reagent. The biosensor constructed by this method has good signal stability and a wide linear range for the detection of tumor markers (PSA). Because the detection platform is simple to prepare and the signal can be collected by taking photos, it could be extended to the measurement of various targets by coupling different recognition probes on the electrode surface.

## Data Availability

Not applicable.

## Supplementary Materials

The following supporting information can be downloaded at: https://www.mdpi.com/article/10.3390/bios13020158/s1. Video S1: Deposition of Au at the cathode of bare GP (cathode)—Au particles modified GP (anode). Video S2: Deposition of Au at the cathode of bare GP (cathode)—Au particles modified GP (anode). Scheme S1: Close bipolar deposition device. Figure S1: Amperometric i-t plots. Figure S2: Effect of water on the dissolution and deposition of Au particles. Figure S3: Effect of 10 mM PBS on the dissolution and deposition of Au. Figure S4: Effects of HCl and different concentrations of PBS on the dissolution and deposition of Au. Figure S5: SEM images of (A) GP, (B) Au-1 film modified GP, and (C) Au-2 film modified GP. Figure S6: XRD patterns of GP substrate, Au-1 film modified GP (Au-1/GP), and Au-2 film modified GP (Au-2/GP). Figure S7: ECL signal obtained on the prepared BPE modified with Au particles at both poles. Figure S8: Reproducibility and stability of the prepared Au modified BPE in ECL measurement. Figure S9: Optical images of Au modified BPE after three consecutive detections. Figure S10: TEM image of SiO_2_ NPs and EIS of different biomolecules modified Au electrodes. Figure S11: Selectivity of the designed biosensor towards thrombin and BSA. Table S1: Electrical conductivity of solutions. Table S2: Performance of the proposed biosensor compared with other biosensors
