# Supplementary material for: Insights into the Mechanism of Bipolar Electrodeposition of Au Films and Its Application in Visual Detection of Prostate Specific Antigens
Source: Biosensors (Basel). 2023 Jan 19;13(2):158. doi: 10.3390/bios13020158 (PMC9953799; doi:10.3390/bios13020158)
Supplement: Supplementary file 1 [file biosensors-13-00158-s001.zip › SI-2022-12-12.pdf]

Insights into the Mechanism of Bipolar Electrodeposition of Au Films and Its Application in Visual Detection of Prostate Specific Antigens

Daoyuan Zhao[a], Yujing Liu[b], Hong Jiang[b], Haijian Yang[a], Huihui Yu[a], Jingtang Qiao[a], Zhiwen Li[a], Bing Jin[a]*, and Meisheng Wu[a]*

[a]Department of Chemistry, College of Sciences, Nanjing Agricultural University, 1 Weigang, Nanjing 210095, P.R. China.

[b]College of Life Sciences, Nanjing Agricultural University, 1 Weigang, Nanjing 210095, P.R. China.

Correspondence: wumeisheng@njau.edu.cn (M. S. Wu.); jinbing@njau.edu.cn (B. Jin)

Contents

1. **Video S1**. Deposition of Au at the cathode of bare GP (cathode)—Au particles modified GP (anode). 10 mM PBS and $HAuCl_4$ were filled into the left and right reservoirs, respectively. A constant voltage of 4.5 V was applied on Pt wires for 5 min. 5 × frame rate.
2. **Video S2**. Deposition of Au at the cathode of bare GP (cathode)—Au particles modified GP (anode). Both reservoirs were filled with $HAuCl_4$. A constant voltage of 4.5 V was applied on Pt wires for 5 min. 10 × frame rate.
3. **Scheme S1** Close bipolar deposition device. (1) Flat PDMS slice, (2) Graphite paper (GP), (3) PDMS slice with two small holes (diameter of 3 mm) on it, and (4) PDMS slice with two large holes (diameter of 7 mm) on it.
4. **Figure S1** Amperometric i-t plots.
5. **Figure S2** Effect of water on the dissolution and deposition of Au particles.
6. **Figure S3** Effect of 10 mM PBS on the dissolution and deposition of Au.
7. **Figure S4** Effects of HCl and different concentrations of PBS on the dissolution and deposition of Au.
8. **Figure S5** SEM images of (A) GP, (B) Au-1 film modified GP, and (C) Au-2 film modified GP.
9. **Figure S6** XRD patterns of GP substrate, Au-1 film modified GP (Au-1/GP), and Au-2 film modified GP (Au-2/GP).

10. Figure S7 ECL signal obtained on the prepared BPE modified with Au particles at both poles.

11. Figure S8 Reproducibility and stability of the prepared Au modified BPE in ECL measurement.

12. Figure S9 Optical images of Au modified BPE after three consecutive detections.

13. Figure S10 TEM image of SiO_2 NPs and EIS of different biomolecules modified Au electrodes.

14. Figure S11 Selectivity of the designed biosensor towards thrombin and BSA.

15. Table S1 Electrical conductivity of solutions.

16. Table S2 Performance of the proposed biosensor compared with other biosensors

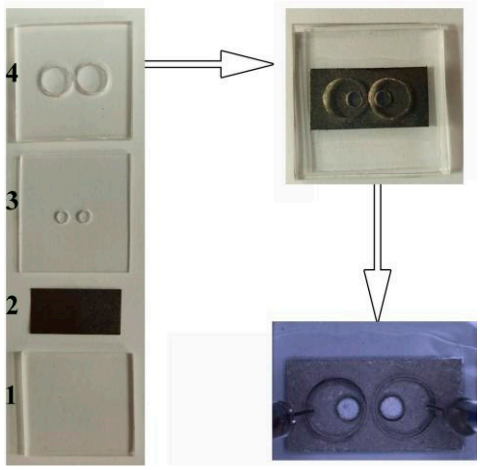

Scheme S1 Closed bipolar deposition device.

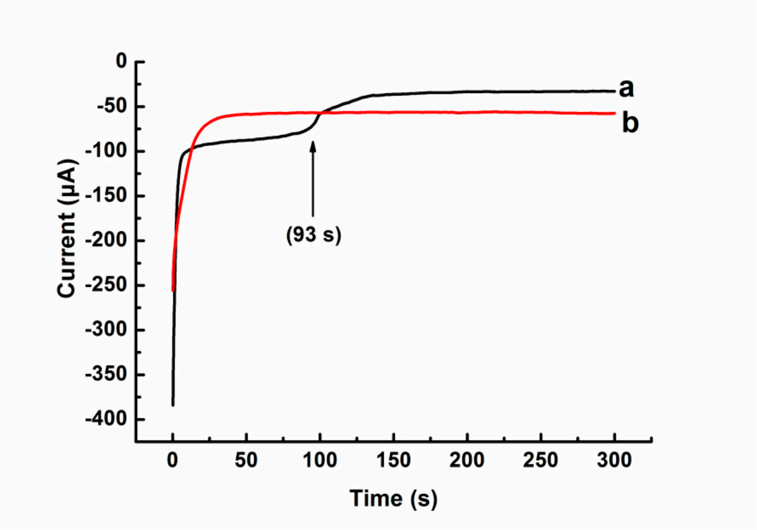

Figure S1 Amperometric i-t plots obtained on bare GP (cathode)-Au modified GP (anode) BPE when the anode was immersed into 10 mM PBS (a) and 5 mM $HAuCl_4$ (b), respectively. The cathode was immersed into 5 mM $HAuCl_4$. The external voltage was 4.5 V.

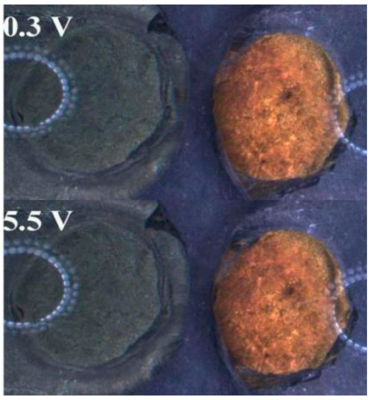

Figure S2 Optical images recorded during the second deposition of Au under linear sweep voltage. The left reservoir was filled with 5 mM $HAuCl_4$ and the right reservoir was filled with water. The voltage was applied from 0 to 5.5 V at a scan rate of 0.005 V/s.

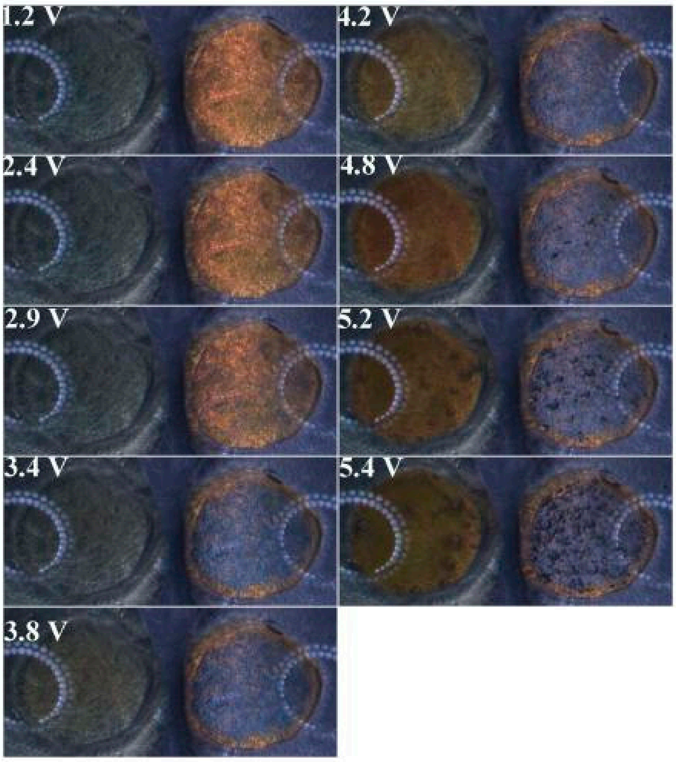

Figure S3 Optical images recorded during the second deposition of Au under linear sweep voltage. The left reservoir was filled with 5 mM $HAuCl_4$ and the right reservoir was filled with 10 mM PBS. The applied voltage was from 0 to 5.5 V at a scan rate of 0.005 V/s.

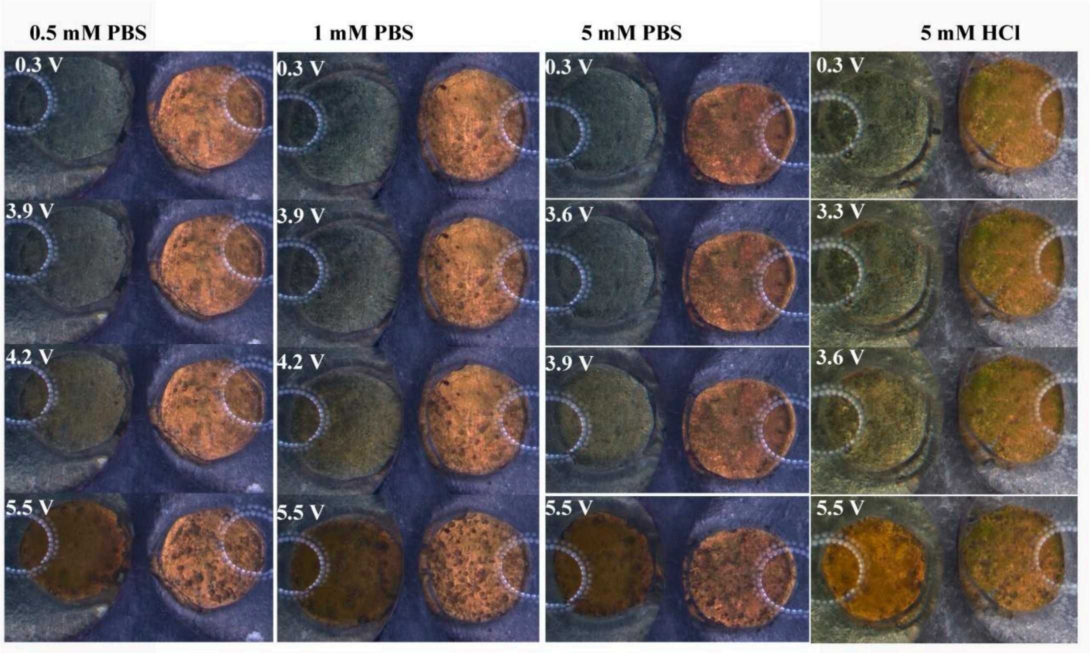

Figure S4 Optical images recorded during the second deposition of Au under linear sweep voltage. The left reservoir was filled with 5 mM $HAuCl_4$ and the right reservoir was filled with 0.5 mM PBS, 1 mM PBS, 5 mM PBS, and 5 mM HCl. The applied voltage was from 0 to 5.5 V at a scan rate of 0.005 V/s.

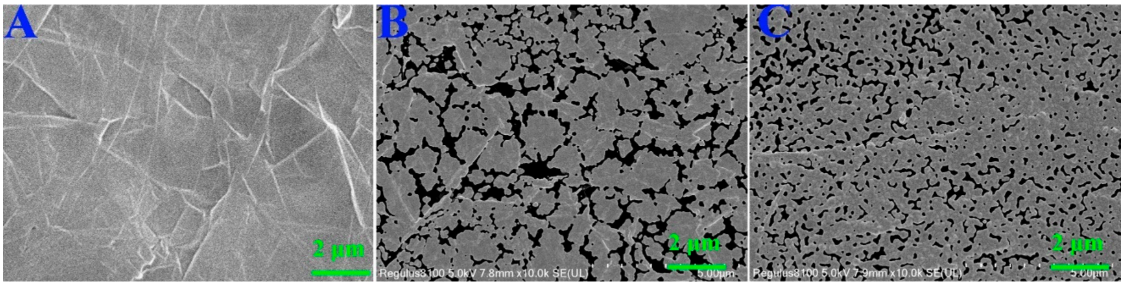

Figure S5 SEM images of (A) GP, (B) Au-1 film modified GP, and (C) Au-2 film modified GP.

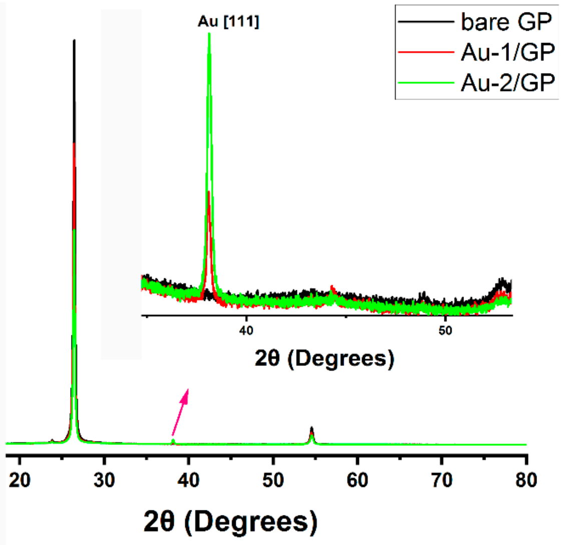

Figure S6 XRD patterns of GP substrate, Au-1 film modified GP (Au-1/GP), and Au-2 film modified GP (Au-2/GP).

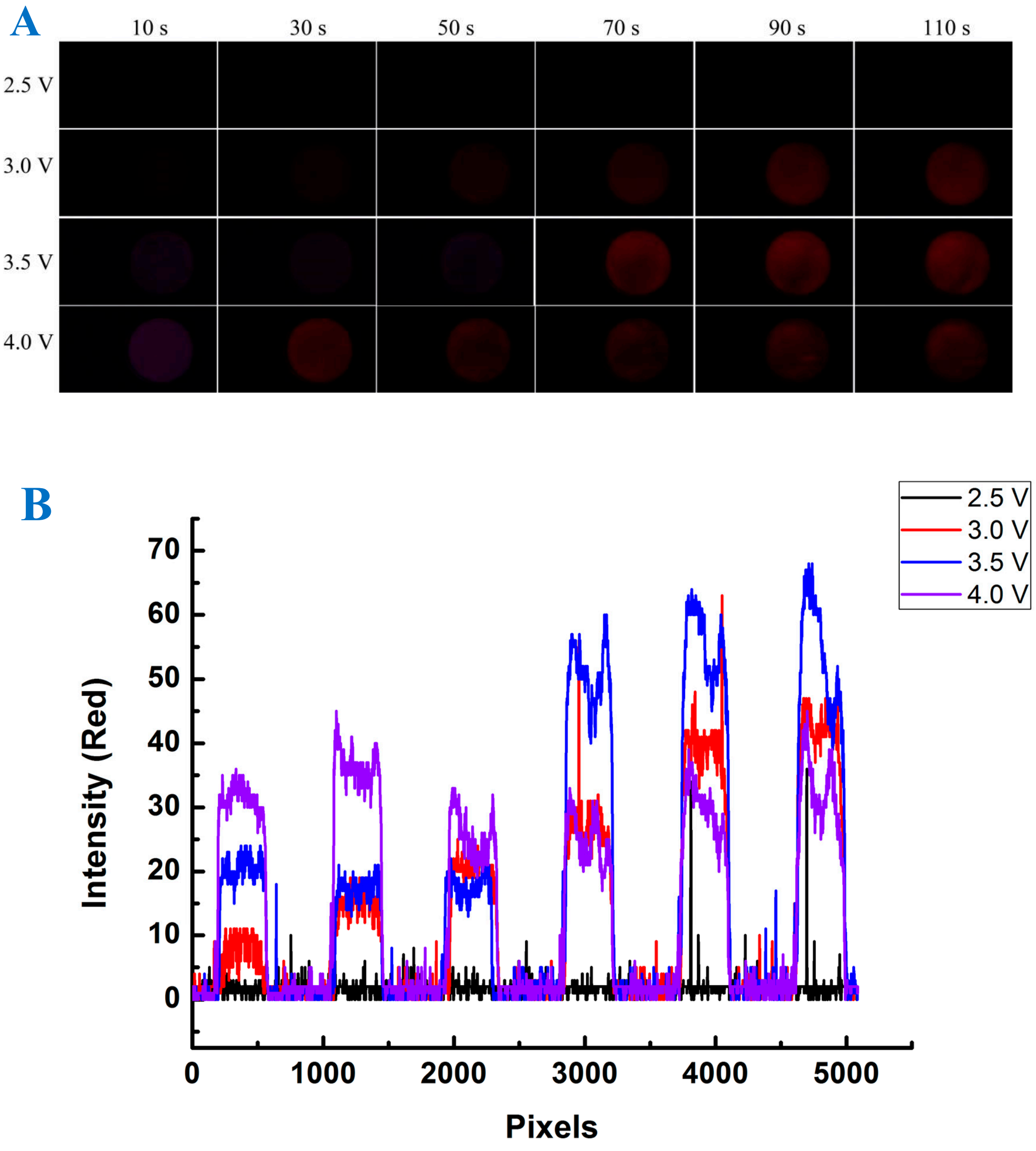

Figure S7 ECL images (A) and corresponding red channel intensity (B) obtained on Au modified (cathode)-Au modified (anode) GP BPE under different external voltages.

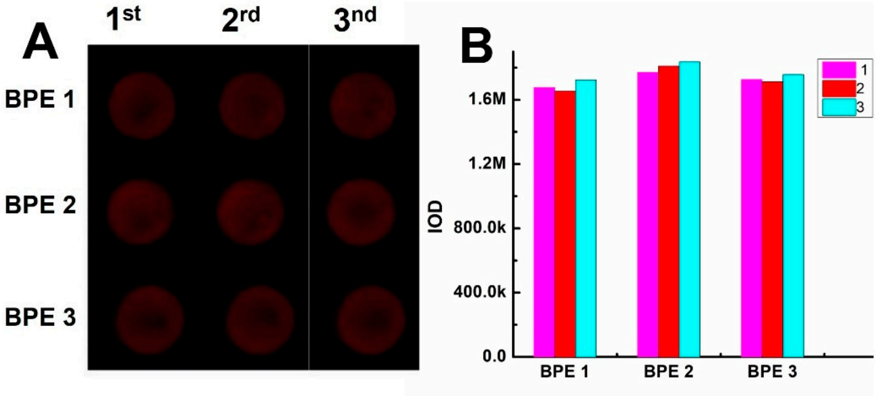

Figure S8 ECL images (A) and corresponding IOD (integrated optical intensity, B) obtained on four Au modified (cathode)-Au modified (anode) GP BPEs for three consecutive measurements.

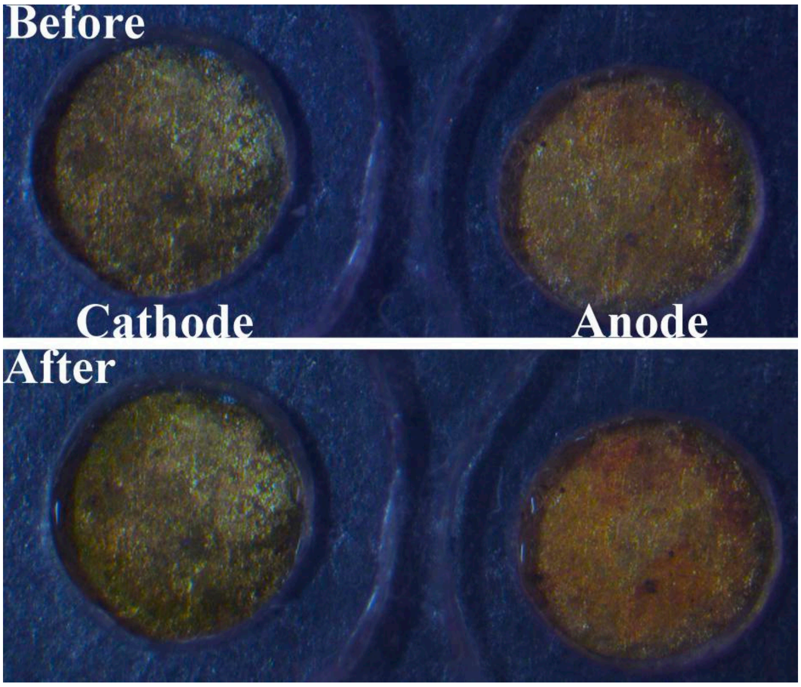

Figure S9 Images of BPE before and after three consecutive ECL detections.

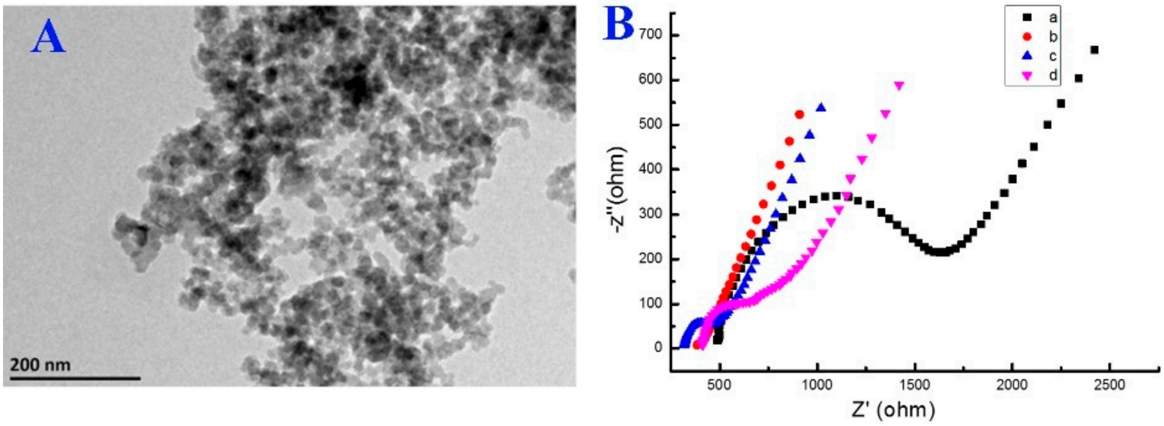

Figure S10 (A) TEM image of SiO_2 NPs. (B) Electrochemical impedance spectrum of various electrodes. Electrode was immersed in 5.0 mM $[Fe(CN)_6]^{3-/4-}$ solution containing 0.1 M KCl. Frequency range: 0.1-10^5 Hz. (a) Bare GP, (b) Ab_1/Au/GP, (c) PSA/Ab_1/Au/GP, (d) Ab_2-SiO_2/PSA/Ab_1/Au/GP.

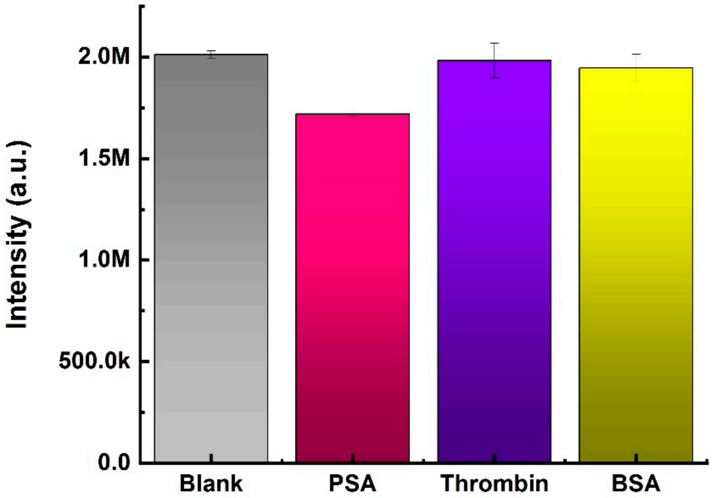

Figure S11 Selectivity of the designed biosensor towards thrombin and BSA. The concentration of PSA was 1 ng/mL. The concentration of thrombin and BSA were 10 ng/mL.

Table S1 Conductivity of solutions

Solution	Conductivity (μS/cm)
10 mM PBS	Out of range
5 mM PBS	8280
5 mM $HAuCl_4$	2340
5 mM HCl	2000
1 mM PBS	1779
0.5 mM PBS	923
H_2O	1.70

Table S2 Performance of the proposed biosensor compared with other biosensors

Analytical method	Linear range (ng/mL)	LOD (ng/mL)	Refs
ECL	1.0 to 1000	0.5	[1]
ECL	0.5 to 50	0.031	[2]
Colorimetric assay	0.1 to 100	0.02	[3]
Electrochromic assay	0.1 to 50	0.03	[4]
Photothermal imaging assay	1 to 200	0.2	[5]
ECL	0.1 to 1000	0.071	Our work

References:

[1] Lu, H.-J., Zhao, W., Xu, J.-J., and Chen, H.-Y. Visual electrochemiluminescence ratiometry on bipolar electrode for bioanalysis, *Biosensors and Bioelectronics* **2018**, *102*, 624-630.

[2] Cao, J.-T., Wang, Y.-L., Zhang, J.-J., Dong, Y.-X., Liu, F.-R., Ren, S.-W., and Liu, Y.-M. Immuno-Electrochemiluminescent Imaging of a Single Cell Based on Functional Nanoprobes of Heterogeneous Ru(bpy)32+@SiO2/Au Nanoparticles, *Analytical chemistry* **2018**, *90*, 10334-10339.

[3] Shayesteh, O. H., and Ghavami, R. A novel label-free colorimetric aptasensor for sensitive determination of PSA biomarker using gold nanoparticles and a cationic polymer in human serum, *Spectrochimica Acta Part A: Molecular and Biomolecular Spectroscopy* **2020**, *226*, 117644.

[4] Lei, Q., Wang, Y., Dong, W., Sun, H., Lv, J., and Li, H. Self-powered electrochromic sensing for visual determination of PSA in serum using PB as an indicator, *Journal of Electroanalytical Chemistry* **2019**, *839*, 108-115.

[5] Lv, S., Zhang, K., and Tang, D. A new visual immunoassay for prostate-specific antigen using near-infrared excited CuxS nanocrystals and imaging on a smartphone, *The Analyst* **2019**, *144*, 3716-3720.
